# Controlled Vapor Phase Growth of Single Crystalline, Two-Dimensional GaSe Crystals with High Photoresponse

**DOI:** 10.1038/srep05497

**Published:** 2014-06-30

**Authors:** Xufan Li, Ming-Wei Lin, Alexander A. Puretzky, Juan C. Idrobo, Cheng Ma, Miaofang Chi, Mina Yoon, Christopher M. Rouleau, Ivan I. Kravchenko, David B. Geohegan, Kai Xiao

**Affiliations:** 1 Center for Nanophase Materials Sciences, Oak Ridge National Laboratory, One Bethel Valley Road, Oak Ridge, TN, 37831, USA

## Abstract

Compared with their bulk counterparts, atomically thin two-dimensional (2D) crystals exhibit new physical properties, and have the potential to enable next-generation electronic and optoelectronic devices. However, controlled synthesis of large uniform monolayer and multi-layer 2D crystals is still challenging. Here, we report the controlled synthesis of 2D GaSe crystals on SiO_2_/Si substrates using a vapor phase deposition method. For the first time, uniform, large (up to ~60 μm in lateral size), single-crystalline, triangular monolayer GaSe crystals were obtained and their structure and orientation were characterized from atomic scale to micrometer scale. The size, density, shape, thickness, and uniformity of the 2D GaSe crystals were shown to be controllable by growth duration, growth region, growth temperature, and argon carrier gas flow rate. The theoretical modeling of the electronic structure and Raman spectroscopy demonstrate a direct-to-indirect bandgap transition and progressive confinement-induced bandgap shifts for 2D GaSe crystals. The 2D GaSe crystals show p-type semiconductor characteristics and high photoresponsivity (~1.7 A/W under white light illumination) comparable to exfoliated GaSe nanosheets. These 2D GaSe crystals are potentially useful for next-generation electronic and optoelectronic devices such as photodetectors and field-effect transistors.

Two-dimensional (2D) nanomaterials with single or few atomic layers exhibit many unique physical and chemical properties when compared to their bulk counterparts^1^,^2^,^3^,^4^. This is easily seen in the case of graphene, which exhibits exotic physical properties never observed from bulk graphite^2^,^5^,^6^,^7^. The controlled synthesis and processing of graphene has opened up new avenues to study the role of dimensionality on the fundamental properties of materials, and has triggered the development of synthesis approaches for other 2D materials^4^. Although graphene shows excellent electrical properties, e.g., high electron and hole mobility (~10^6^ cm^2^/Vs), its zero bandgap energy limits its applications in logic electronics and field-effect transistors (FETs)^4^,^7^. In order to overcome these limitations, a variety of 2D materials beyond graphene with different bandgaps have been synthesized in recent years^4^,^8^, including insulating *h*-BN^9^,^10^ and semiconducting layered transition metal dichalcogenides, e.g., MoS_2_^9^,^11^,^12^,^13^,^14^, WS_2_^15^,^16^, and WSe_2_^17^,^18^. These materials show many unique optical and electrical properties, e.g., indirect-to-direct bandgap transitions and valley polarization^19^,^20^, and enrich the number of building blocks that may be used for next-generation electronic and optoelectronic devices.

Although most research has focused on 2D transition metal dichalcogenides, recently 2D layered metal monochalcogenides, e.g., GaS, GaSe, and InSe, have attracted increasing interest^21^,^22^,^23^,^24^,^25^. Recent studies on these 2D metal monochalcogenides indicate that they have significantly different electronic and optoelectronic properties from transition metal dichalcogenides^21^,^22^,^23^,^24^,^25^. Gallium selenide (GaSe) is a typical layered metal mono-chalcogenide, which has a direct bandgap energy of ~2 eV^26^ and generally shows p-type behavior^27^,^28^. Bulk GaSe crystals can be constructed conceptually by vertically stacking single layers which are held together by van der Waals forces, with different stacking modes giving rise to different bulk crystal structures, i.e., *β*-GaSe, *ε*-GaSe, *γ*-GaSe, and *δ*-GaSe^29^. A single layer of GaSe is composed of covalently bonded Se−Ga−Ga−Se atoms, with a lattice constant of 0.374 nm and D*_3h_* symmetry^29^,^30^. Bulk GaSe shows many interesting electrical and optical properties, e.g., anisotropic Hall-mobility^31^, electron-hole liquid behavior^32^, and up-conversion luminescence^33^, and has been widely used in the fields of optoelectronics, nonlinear optics, and terahertz radiation^34^,^35^,^36^. Recently, both theoretical and experimental work have revealed many new properties as bulk GaSe has been thinned systematically to 2D crystals. Theoretical studies have predicted that the bandgap of GaSe may be widely tuned by varying the numbers of layer in the crystal or by the inducing mechanical strain^37^,^38^,^39^. In addition, the low formation energy and band edge positions of monolayer GaSe makes it a suitable photocatalyst for solar water-splitting^40^. GaSe nanosheets with several atomic layers have been fabricated through mechanical exfoliation^22^,^23^, and show good optical and electrical performance as photodetectors and FETs, revealing the great potential of this 2D nanomaterial for such devices.

In order to realize practical uses of 2D materials-based devices, it is essential to synthesize large-area, high-quality 2D crystals with controlled layer numbers on various insulating and conducting substrates. To this end, 2D materials are generally fabricated by mechanical or chemical exfoliation from their layered bulk counterparts, or by vapor phase deposition, which includes thermal evaporation and chemical vapor deposition (CVD). Mechanically exfoliated 2D crystals generally have higher quality, but they are limited in size (usually up to tens of microns) and the thickness is not uniform and controllable. The vapor phase deposition method, on the other hand, has been widely used to grow large-area graphene and h-BN nanosheets^10^,^41^, as well as large, single crystal, monolayer semiconducting 2D materials such as MoS_2_ and WS_2_^12^,^13^,^14^,^15^,^16^. Although recently few-layer GaSe 2D crystals (up to ~20 μm in lateral size) have been synthesized on SiO_2_/Si substrates through vapor-phase mass transport, the use of nucleation seeds and the growth in a sealed quartz tube makes control of the uniformity and layer number of 2D GaSe crystals difficult^24^. To date, it is still a challenge to synthesize large and uniform 2D GaSe crystals with controllable layer numbers.

Here, we synthesized monolayer and multi-layer 2D GaSe crystals directly on SiO_2_/Si substrates using a vapor phase deposition method, in which large (up to ~60 μm in lateral size), uniform, single-crystalline monolayer GaSe crystals were obtained for the first time. The structure and orientation of 2D GaSe crystals are characterized from the atomic scale to micrometer scale by using scanning transmission electron microscopy (STEM) and dark-field transmission electron microscopy (DF-TEM). The well-defined 2D crystals were controllable by tuning growth conditions. The Raman spectra of the 2D GaSe crystals change with their layer numbers in both peak positions and intensities. The Raman and theoretical modeling of the electronic structure of 2D GaSe crystals demonstrate a direct-to-indirect bandgap transition and a progressive confinement-induced bandgap shift, which are significantly different from the widely studied transitional metal dichalcogenides such as MoS_2_. These 2D GaSe crystals show a high photoresponse with white light illumination, and great potential for next-generation electronic and optoelectronic devices.

## Results and Discussion

**Figure 1:**
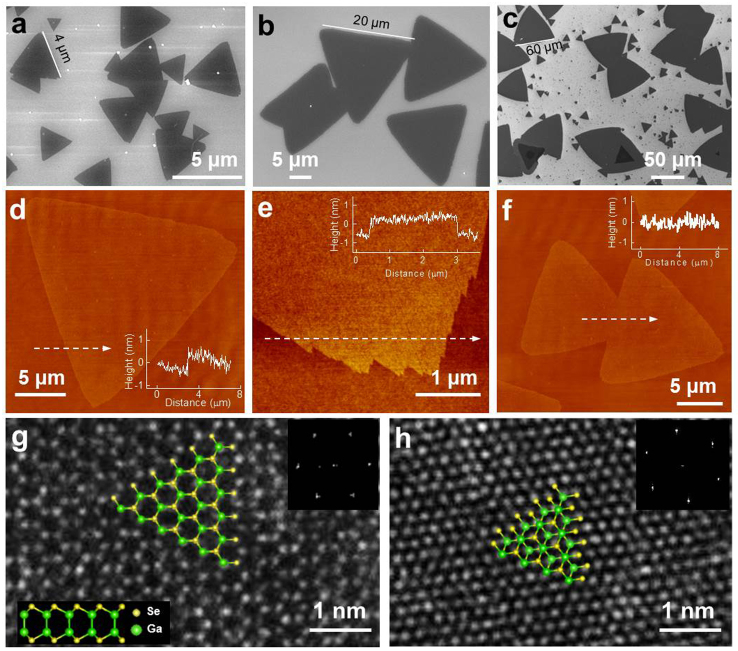
Growth of 2D GaSe crystals. (a–c) SEM images of monolayer triangular GaSe crystals grown for 2 min (a), 5 min (b), and 10 min (c). (d–f) AFM images of monolayer GaSe crystals. Insets are line profiles in the direction of the dashed arrows. Images (d) and (f) show an individual and two merged triangular monolayer flakes, respectively, while image (e) shows an enlarged view of the tip of the triangle. (g) High-resolution ADF-STEM image of monolayer GaSe. The lattice is composed of hexagonal rings of gallium and selenium atoms. Top and side views of monolayer GaSe structure are overlaid. Inset is the corresponding FFT image. (h) High-resolution ADF-STEM image of multi-layer GaSe. Top view of multi-layer GaSe structure with ε-type stacking is overlaid. Inset is the corresponding FFT image.

The 2D GaSe crystals were synthesized in a tube furnace system equipped with a 1″ diameter quartz tube (Figure S1). A mixture of bulk GaSe (Figure S2) and Ga_2_Se_3_ powders (molar ratio: ~50:1) was thermally evaporated at 750°C under a pressure of 30 Torr and an argon carrier gas flow rate of 50–100 sccm (standard cubic centimeter per minute). The use of a small amount of Ga_2_Se_3_ provides sufficient Se for growing regularly-shaped triangle monolayer flakes, which will be discussed in detail below. Compared with previous approaches to synthesize 2D GaSe through vapor-phase mass transport within sealed tubes^24^, the precise control over the gas flow rate and reaction chamber pressure in this study allowed reproducible and controllable correlation between growth conditions and resulting nanostructure. The 2D products were deposited on SiO_2_ (~300 nm)/Si substrates (See Methods for detailed synthesis process). Figure 1a–c show typical scanning electron microscopy (SEM) images of the 2D products synthesized at the growth temperature from ~710 to ~720°C, with an argon gas flow rate of 50 sccm, and growth times of 2 min (Figure 1a), 5 min (Figure 1b), and 10 min (Figure 1c). The products are triangular flakes, some of which merge together to form irregularly-shaped islands. The triangular flakes are composed of Ga and Se with an atomic ratio of 1:1 (GaSe) as determined by energy-dispersive x-ray spectroscopy (EDS) (Figure S3; the bright, small particles on the flakes are Se nanoparticles deposited during the synthesis, and can be removed by heat treatment at 300°C in vacuum). The lateral size of the triangular flakes increases with increasing growth time, from up to ~4 μm for 2 min (Figure 1a), to ~20 μm for 5 min (Figure 1b), and to ~60 μm for 10 min (Figure 1c). The average lateral size of the triangular flakes depending on growth times are presented in Figure S4. Except for only a few thicker patches with darker contrast, the largely uniform contrast in the SEM images reveal that most of the GaSe domains have uniform thickness (Figure 1c).

The detailed morphology and thickness of the GaSe flakes were characterized by atomic force microscopy (AFM). Figure 1d shows the AFM image of an individual triangular flake, which is equilateral with sharp and smooth edges. A dashed-line arrow across the edge shows that the thickness of the flake is ~0.8 nm, corresponding to a single atomic layer of GaSe^38^. Statistics from a series of SEM images covering the whole substrate indicates that more than 90% of the GaSe domain is monolayer. The result demonstrates that large, uniform monolayer GaSe crystals were grown on the SiO_2_/Si substrate for the first time (the darker domains shown in Figure 1c correspond to multi-layer flakes, which will be discussed in detail later (Figure 3)). An enlarged AFM image indicates that the area around the tip of the triangle has more irregularities when compared with the side of the triangle (Figure 1e). Such jagged edges were also observed in other 2D monolayer materials^12^,^16^. Figure 1f shows the AFM image of two merged monolayer GaSe flakes. This domain is uniform in thickness, indicating that when the two monolayer GaSe flakes made contact, they merged instead of one overgrowing the other.

The crystal structures of our vapor-phase grown 2D GaSe were characterized using aberration-corrected STEM and electron diffraction. The 2D samples for structural characterizations were grown directly on amorphous silicon films (5 nm in thickness) supported by a silicon TEM grid under the same conditions as described above. The atomic structure of the monolayer GaSe flake is shown with annular aberration-corrected dark-field STEM (ADF-STEM) imaging in Figure 1g. Hexagonal rings composed of Ga and Se atoms are clearly visible, in agreement with the hexagonal structure of monolayer GaSe (as indicated by the top- and side-view schematics in Figure 1g). The distance between two in-plane adjacent Ga (or Se) atoms is ~0.38 nm, matching the lattice parameters of the *a*-*b* plane (0.375 nm) in GaSe crystals^29^, but it is hard to distinguish Ga and Se atoms in the ADF-STEM image due to their similar atomic numbers^13^, and because the GaSe flakes were imaged on top of amorphous silicon membranes, which results in a reduction of contrast. The corresponding Fast Fourier Transforms (FFT) of the image (inset of Figure 1g) confirms the hexagonal structure of the monolayer GaSe. In contrast to the hexagonal-ring structure of monolayer GaSe crystals, multi-layer GaSe flakes generally show a close-packed structure along *c*-axis as shown in Figure 1h. Such a structure indicates that the stacking of the multi-layer crystal can be *ε*-, *γ*-, or *δ*-type, but not *β*-type, as indicated by top-view schematic in Figure 1h.

**Figure 2:**
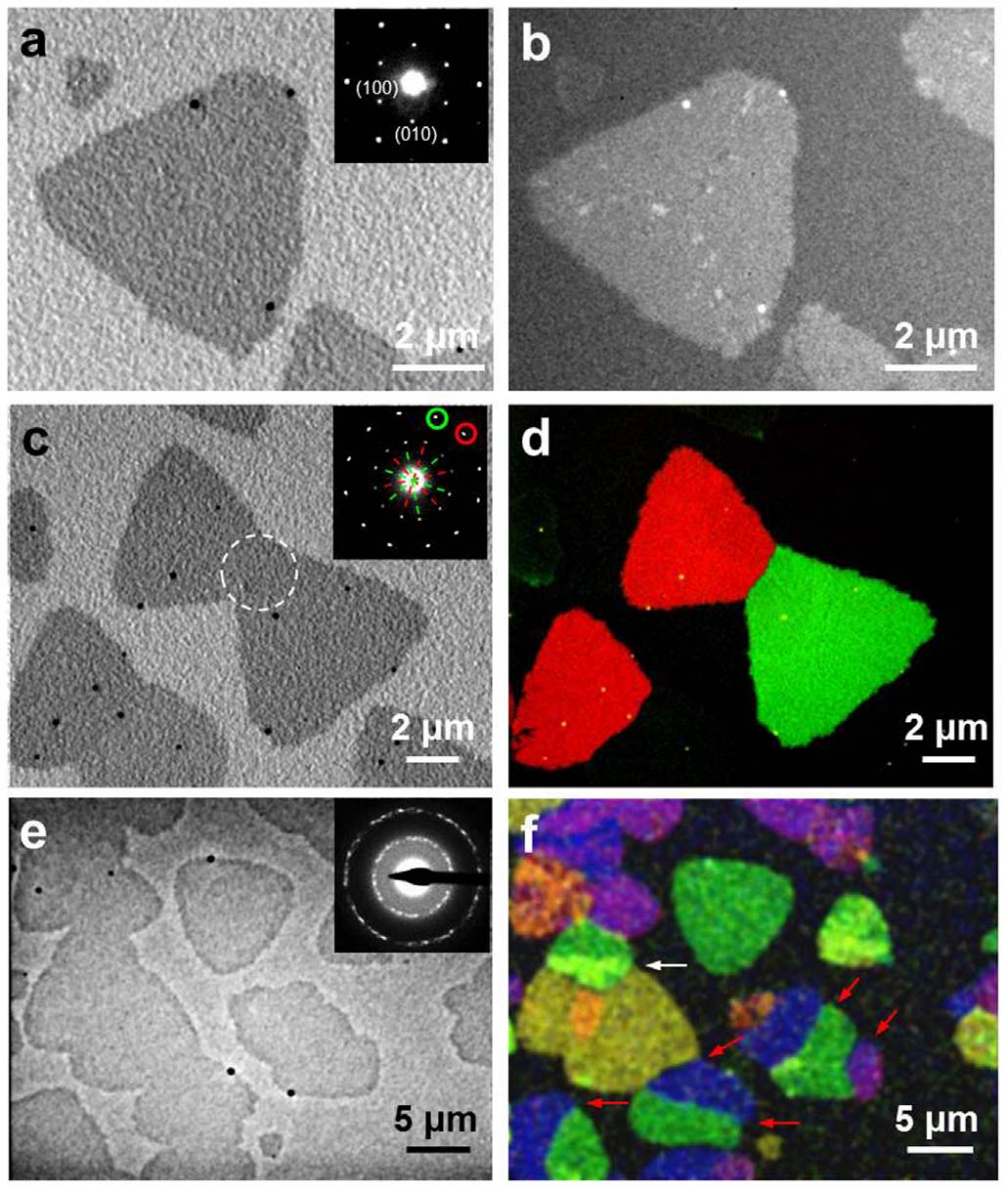
Grain structures in monolayer GaSe crystals. (a) Bright-field TEM image of a single monolayer triangular flake. Inset is the SAED pattern of the flake, showing a single set of spots in a hexagonal pattern. (b) DF-TEM image of the flake in (a). (c) Bright field TEM image showing two monolayer triangular flakes merging together. Inset is the SAED pattern obtained from the common area of the two flakes as indicated by a dashed circle. The pattern shows two sets of spots in a hexagonal pattern (indicated by red and green dashed-lines, respectively) with orientated ~30° apart. (d) Color-coded overlay of DF-TEM images corresponding to the red- and green-circled diffraction spots in the inset of (c). (e) Bright-field TEM image of an area containing both monolayer triangular flakes and large islands of merged flakes. Inset is the electron diffraction pattern obtained from the whole area in (e). (f) Color-coded overlay of DF-TEM image of the area in (e). The overlapped crystal grains are indicated by the white arrows and the clear grain boundaries are indicated by the red arrows.

The crystallinity, grain orientation, and grain boundary of 2D monolayer GaSe flakes were studied by selected-area electron diffraction (SAED) and DF-TEM. Figure 2a shows the bright-field TEM (BF-TEM) image of a single monolayer triangular flake. Note that the triangles grown on the 5 nm-thick amorphous silicon film are not as sharp and smooth as those grown on SiO_2_/Si substrates (Figure 1), indicating that the substrate may play an important role in determining the shape of the monolayer flakes. The SAED pattern obtained from the flake in Figure 2a shows only one set of six-fold symmetry diffraction spots (inset of Figure 2a), indicating that the flake is single crystal with a hexagonal crystal structure. The corresponding DF-TEM image further confirms the single-crystal nature of the triangle GaSe flake (Figure 2b). The BF-TEM image in Figure 2c highlights a pair of merged monolayer triangle flakes with a crystal misorientation of ~30° as indicated by two sets of hexagonal diffraction patterns in the inset of Figure 2c. The angle-resolved, false color DF-TEM image (Figure 2d), made by overlaying two color-coded DF-TEM images acquired from the red- and green-circled diffraction spots, respectively, demonstrates that the intersection of the two grains forms a very sharp boundary. Figure 2e shows the BF-TEM image of a region containing both isolated triangle flakes and islands of merged flakes. The electron diffraction pattern (inset of Figure 2e) acquired from all the flakes in Figure 2e shows typical polycrystalline rings, indicating the flakes have different crystallographic orientations. This can be visualized in the false color DF-TEM image in Figure 2f, wherein different crystallographic orientations shown in the electron diffraction pattern in Figure 2e correspond to different colors. The result indicates that the irregularly-shaped islands are comprised of single crystal flakes having different orientations, with most of them connected to each other by grain boundaries (indicated by red arrows in Figure 2f), and only a few overlapping to form bilayer regions (indicated by a white arrow in Figure 2f).

**Figure 3:**
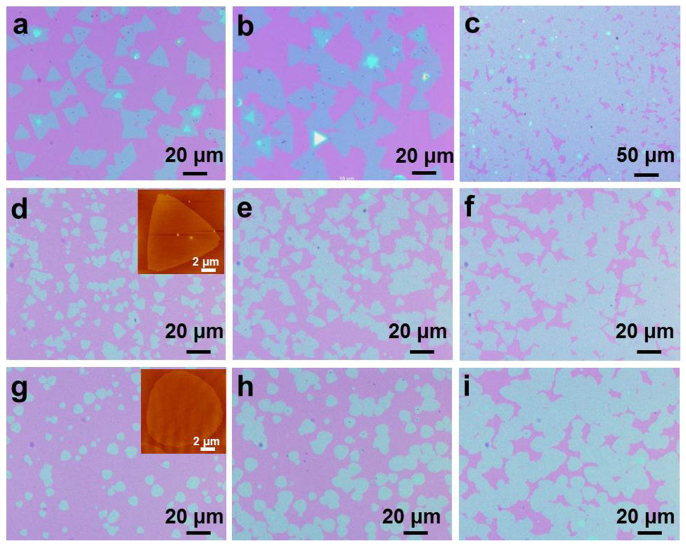
Influence of growth conditions on monolayer GaSe crystals. Optical micrographs of 2D monolayer GaSe crystals synthesized at a growth temperature of (a–c) ~710–720°C, (d–f) ~700–710°C, and (g–i) ~660–670°C, with an argon gas flow rate of 50 sccm and a growth time of 5 min. The images were obtained near the downstream side (a, d, g), middle (b, e, h), and upstream side (c, f, i) of the substrates. Insets of (d) and (g) are AFM images of individual crystals.

In our vapor-phase growth process, the size, density, shape, thickness, and uniformity of the resulting 2D GaSe crystals were strongly influenced by the growth region in the furnace, growth temperature, and argon carrier gas flow rate. Figures 3a–c show optical micrographs of the monolayer GaSe synthesized at growth temperatures from ~710 to ~720°C, with an argon gas flow rate of 50 sccm, and a growth time of 5 min. The three images were obtained from three different regions on the SiO_2_/Si substrate, i.e., close to the downstream side (right side of the substrate as shown in Figure S1) (Figure 3a), around the middle (Figure 3b), and close to the upstream side (left side of the substrate as shown in Figure S1) (Figure 3c). The images clearly show that for growth regions closer to the upstream side of the furnace, the sizes of the triangular flakes increased and more islands of merged triangles were formed, with all the flakes merging into a continuous film in the region closest to the upstream side of the substrate. Such changes in size and density of the monolayer GaSe crystals with different growth regions may be caused by the temperature gradient and the change in diffusion flux of the source along the substrate^42^. In all cases studied, if the substrate was placed at a lower temperature region, i.e., ~700–710°C (Figure 3d–f) and ~660–670°C (Figure 3g–i), the synthesized monolayer GaSe crystals followed the same trend, merging into a continuous layer in the upstream region of the substrate due to higher nucleation density. Note that the gas flow in our growth tube is laminar – i.e., Reynold's number ~2.2 (see Supporting Information for detailed calculation)^42^ – and this, taken with the results above, indicate the growth of monolayer GaSe was controlled mainly by a diffusion process through a boundary layer, the thickness of which plays an important role in controlling the diffusion flux^42^. In the present case, the diffusion flux on the substrate is inversely proportional to the distance from the side of the substrate closest to the source materials^42^, and a since higher diffusion flux generates more nucleation sites and favors higher growth rate, the GaSe flakes grown near the upstream side of the substrate (closer to the source) show larger size and higher density than those near the downstream side.

It has already been shown by structural characterization that the shape of monolayer GaSe flakes may be influenced by the growth substrate. In fact, the shape of monolayer GaSe is also sensitive to growth temperature. As already demonstrated by the results in Figure 1 and Figures 3a–c, equilateral triangular monolayer flakes with smooth and straight edges were grown at ~710–720°C. However, when the growth temperature was reduced (with all other growth conditions unchanged) to ~700–710°C, equilateral triangular monolayer GaSe flakes were still obtained (Figure 3d–f), but with edges that are not as linear as those grown at ~710–720°C. Further decreases in the growth temperature, i.e., at ~660–670°C, resulted in monolayer GaSe flakes that were almost round (Figure 3g–i), and the change of the shape at different growth temperatures might be explained by the minimum energy shape of monolayer GaSe^43^. Based on the 3-fold symmetry of a single atomic layer of GaSe^29^,^30^, the minimum energy shape is expected to be a triangle^43^. However, at low growth temperatures depositing GaSe molecules may be captured immediately when encountering a growing nucleus, and lack sufficient energy to desorb and move to energetically preferred location. As deposition and surface diffusion are random, rounded flakes are necessarily formed. In contrast, at higher growth temperature the depositing GaSe molecules have enough mobility to desorb and move from random, high energy positions to minimum energy positions, thereby favoring triangular flakes. As shown in Figure S5, rounded monolayer GaSe flakes were also formed when only GaSe was used as the source material (without the small amount of Ga_2_Se_3_ powders), and may be explained by considering the concentration of vapor-phase reactant species in the growth region. During the thermal evaporation process, bulk GaSe decomposes into vapor-phase Ga_2_Se and Se_2_, which then diffuse to the substrate with different velocities and mean free paths^44^ (this could be the reason for the deposition of Se nanoparticles on 2D GaSe flakes as observed in SEM images). The lack of one species may impede the growth of the 2D crystals as seen in previous reports wherein GaSe and MoS_2_ flakes grown by vapor-phase deposition were transformed from regular triangles to truncated triangles and even to hexagons as the concentration of selenium or sulfur was reduced^14^,^24^. Therefore, it is possible in the present case that the concentration of Se_2_ is lower than Ga_2_Se in the growth region when only GaSe is used, and the addition of Ga_2_Se_3_ increases the Se_2_ concentration, resulting in regularly-shaped triangle flakes. It should be noted that the amount of Ga_2_Se_3_ must be well controlled, with the best molar ratio of GaSe/Ga_2_Se_3_ being ~50/1 (larger amounts of Ga_2_Se_3_ resulted in the formation of Ga_2_Se_3_ crystals on the substrate).

**Figure 4:**
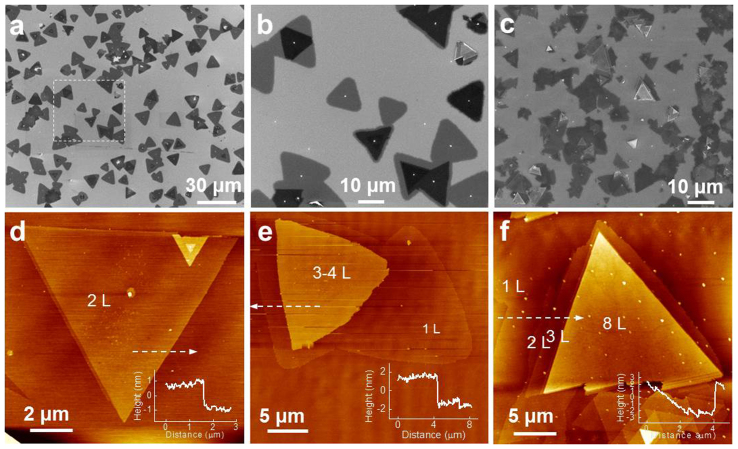
Multi-layer 2D GaSe crystals. (a–c) SEM images of GaSe crystals grown with an 80 sccm argon flow for 5 min. Images (a) and (b) were obtained from the region close to the downstream side of the substrate. The flakes with lighter contrast are monolayer, while darker flakes indicate additional layers grown on monolayer flakes. (b) is the enlarged image of the area contained in the dashed square in (a). Image (c) shows thicker multi-layer GaSe crystals on a continuous monolayer GaSe film grown in the region close to the upstream side of the substrate. (d–f) AFM images of multi-layer GaSe crystals. Insets are line profiles along the dashed arrows.

The argon gas flow rate played an important role in controlling the number of layers in the 2D GaSe flakes. The results in Figure 1 and Figures 3a–c have already shown that uniform monolayer GaSe flakes (>90% of all the GaSe domains) were grown with an argon gas flow rate of 50 sccm. However, the quantity of multi-layer flakes can be increased deliberately by increasing the argon gas flow rate. Figures 4a–c show SEM images of 2D GaSe crystals grown with 80 sccm argon gas flow and a growth time of 5 min. Isolated and merged monolayer (in lighter contrast) and multi-layer (in darker contrast) flakes are observed near the downstream side of the substrate (Figure 4a, b). The monolayer domains in this region decrease to ~60% of all the GaSe domains, and the multi-layer flakes generally have 2–4 layers (2–4 L) as determined by AFM (Figure 4d, e). As shown in the SEM images (Figure 4a, b), the size of the multi-layer triangular flakes are generally smaller than the underlying monolayer ones (multi-layer flakes truncated as they grew to the edges of monolayer flakes can be observed, as shown in Figure 4b). With the growth region moving towards the upstream side of the substrate, a higher density of thicker (up to ~8–15 L) multi-layer GaSe flakes were grown on what appears to be a continuous film of monolayer GaSe (Figure 4c, f). These results indicate that the growth of multi-layer GaSe crystals is likely governed by a ‘layer-plus-island’ growth mode^45^, and a higher argon carrier gas flow rate, which results in a higher overall diffusion flux of the source^42^, favors the growth of multi-layer flakes. Indeed, further increasing the argon gas flow rate, i.e., to 100 sccm, led to more and much thicker (up to ~55 L) multi-layer GaSe flakes on continuous monolayer films (Figure S6). However, when the argon gas flow rate was lowered, i.e., to 20 sccm, no in-plane (horizontal to the substrate) monolayer and multi-layer GaSe flakes were obtained. Instead, multi-layer GaSe flakes grew vertically on the substrate, forming ‘flower-like’ structures (Figure S7).

**Figure 5:**
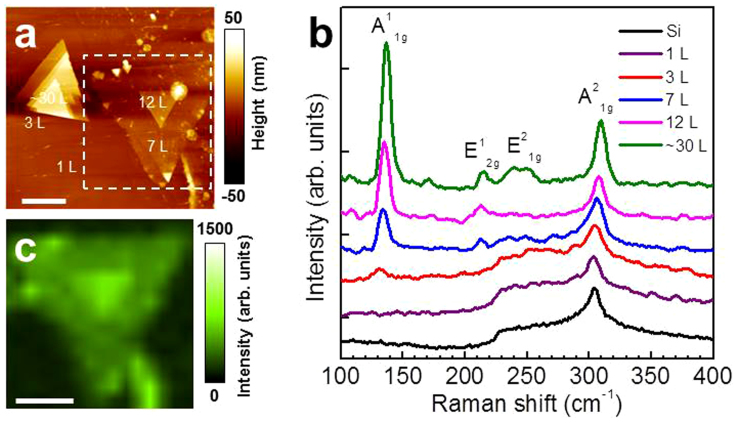
Optical properties of 2D GaSe. (a) AFM image of 2D GaSe crystals with different layer numbers (from 1 to ~30 L). The scale bar is 5 μm. (b) Raman spectra (532 nm laser excitation) of 2D GaSe crystals with 1, 3, 7, 12, and ~30 L as indicated in (a) and a bare substrate. Note that the spectra were offset for clarity. (c) Raman mapping of the crystals included in the dashed square in (a) by monitoring A^1^_1g_ peak in the Raman spectra. The scale bar is 5 μm.

One of the most prominent characteristics of 2D materials is their physical properties depending strongly on layer number. In this work, we studied the optical properties of our 2D GaSe crystals with different layer numbers using Raman spectroscopy. Figure 5a shows the AFM image of 2D multi-layer GaSe crystals with different layer numbers grown on a continuous monolayer film (see also Figure S8 with line profiles determining layer numbers). The Raman spectrum (measured with 532 nm laser excitation) of a GaSe crystal with ~30 L shows peaks at ~136.7 cm^−1^, 215.6 cm^−1^, 244.1 cm^−1^, and 309.3 cm^−1^, corresponding to the A^1^_1g_, E^1^_2g_, E^2^_1g_, and A^2^_1g_ vibration mode of GaSe, respectively (Figure 5b, green curve)^46^. As shown in Figure 5b, the intensity of these Raman peaks decreases with the reduction of layer numbers as shown by the Raman spectra of GaSe crystals with 12 L (pink curve), 7 L (blue curve), 3 L (red curve) and 1 L (purple curve). Note that the Raman spectrum of monolayer GaSe (1 L) is almost the same as that of Si from the SiO_2_/Si substrate (Figure 5b, black curve). This phenomenon was also observed in exfoliated GaSe nanosheets^22^,^47^. In addition to the intensity, the Raman peaks also show shifts with the change in layer numbers. The A^1^_1g_ peak, corresponding to the out-of-plane mode, is located at ~136.7 cm^−1^ for the GaSe crystal with ~30 L, at ~135.3 cm^−1^ for 12 L, ~133.8 cm^−1^ for 7 L, and ~131.1 cm^−1^ for 3 L (hardly observed for 1 L). The red-shift of the peak with the decrease in layer numbers is probably due to the decrease in inter-layer interaction. Another peak associated with the out-of-plane mode, the A^2^_1g_ peak, also shows red-shift from ~309.3 cm^−1^ for the ~30 L-crystal to ~306.5 cm^−1^ for the 7 L-crystal; however, it is hard to study the shift of A^2^_1g_ peak for thinner GaSe flakes (e.g., 1 L and 3 L) because of the increasing Raman signal from the substrate, e.g., the peak at ~303.3 cm^−1^ from Si, which overlaps with the spectra of GaSe. A Raman map of the 7 L- and 12 L-GaSe crystals (included in the dashed square in Figure 4a) was obtained by monitoring the A^1^_1g_ peak (Figure 5c), and except for some brighter spots on the edge of the flakes, which originate from small, thicker crystals as shown in Figure 5a, the intensities were uniform within the terrace region, suggesting good quality crystals.

**Figure 6:**
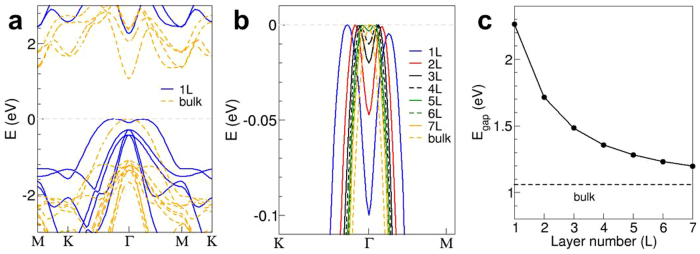
Electronic band structures of 2D GaSe from theoretical calculations. (a) Energy band plots of monolayer and bulk GaSe along the high symmetry k-points. (b) Energy bands near the valence band maximum. (c) Bandgap energy as a function of layer numbers.

To understand the effect of dimensional confinement on the electronic properties of 2D GaSe crystals, theoretical calculation of the electronic band structures was performed. The electronic band structures of 2D GaSe crystals were investigated using a highly accurate, all-electron first-principles quantum mechanical calculation code (FHI-aims^48^). The exchange-correlation potential of the Perdew-Burke-Ernzerhof (PBE) version of the generalized-gradient approximation (GGA)^49^ was used. Figure 6a shows the electronic band structures of monolayer GaSe and its *ε*-type bulk counterpart, with respect to their valence band maximum (VBM) (see Supporting Information for detailed calculation process). Both the VBM and the conduction band minimum (CBM) are located at the Γ point for bulk, which means that bulk *ε*-GaSe has a direct bandgap, which agrees with previous calculations and experimental result^26^,^40^,^50^. However, with decreasing layer number (<7 L), as shown in Figure 6b, the VBM splits in a symmetric way along the Γ point, and a progressive confinement-induced bandgap shift for 2D crystals occurs, indicating that these crystals have an indirect bandgap and its energy increases with decreasing layer numbers (Figure 6c). This is consistent with the blue-shifted emission bands of 2D GaSe crystals observed in exfoliated 2D GaSe nanosheets^23^. The theory-predicted direct-to-indirect bandgap transition with decreasing layer number of 2D GaSe also contribute to the decreasing Raman intensity due to the suppressed inter-layer electron orbital coupling^51^. These findings demonstrate that 2D GaSe crystals are significantly different with the widely studied MoS_2_, which has an indirect-to-direct bandgap transition in monolayer^11^,^12^,^13^,^14^,^19^. Moreover, the energy difference between the direct gap and indirect gap for monolayer GaSe is so small that electrons can easily move between the minima with a small amount of thermal energy.

**Figure 7:**
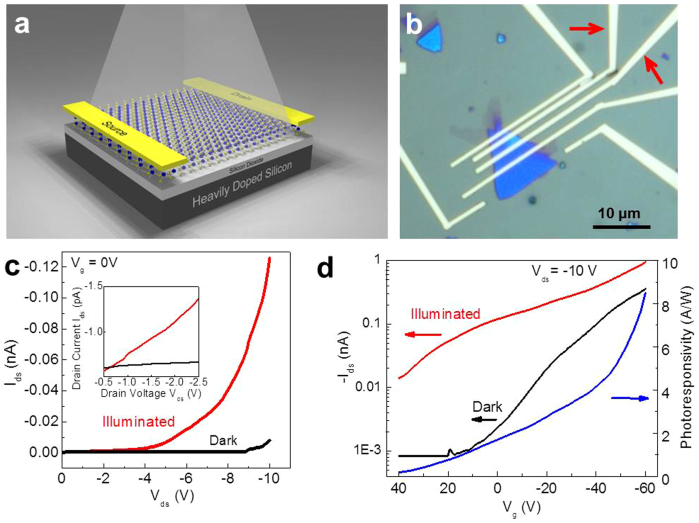
Photoconducting properties of 2D GaSe crystals. (a) Illustration of a 2D GaSe-based device illuminated with white light. (b) Optical image of a 2D GaSe device with patterned electrodes. The red arrows indicate the two electrodes used for measurements. (c) I_ds_-V_ds_ characteristics of the 2D GaSe device in the dark (black curve) and with white light illumination (power density: 1.2 mW/cm^2^) (red curve). (d) The transfer characteristics of the 2D GaSe FET with V_ds_ = −10 V in the dark (black curve) and with white light illumination (red curve). Calculated responsivity as function of the V_g_ also shown (blue curve).

The large scale vapor phase growth of 2D GaSe crystals directly on device-compatible SiO_2_ substrates is favorable for conventional lithographic processes since this method does not need an extra transfer step, a step that may cause damage and contamination to the mechanically delicate 2D crystals. We used a standard e-beam photolithography process to make devices on 2D GaSe crystals grown on SiO_2_/(p^++^)Si substrates as illustrated in Figure 7a. Unfortunately, it is hard to get electrical signals from devices fabricated on monolayer GaSe. Figure 7b shows the optical micrograph of a few-layer GaSe-based device with patterned electrodes (see also Figure S9a). Electrical characterization was carried out both in darkness and under the illumination (white light, 1.2 mW/cm^2^). The current was measured using two electrodes as indicated by the red arrows in Figure 7b, and the effective area of this 2D GaSe-based device was ~5.73 μm^2^. Figure 7c shows the drain-source (I_ds_-V_ds_) characteristic of the device in the dark (black curve) and under white light illumination (red curve), and the I_ds_-V_ds_ curves are linear (Inset of Figure 7c) and symmetric for small bias voltages (see Figure S9b), indicating Ohmic contacts. At a V_ds_ of −10 V, the I_ds_ of the device increased by three orders of magnitude (relative to the dark current) when exposed to white light illumination, indicating good photoresponse. Note that the photocurrent (I_ph_) also increased with bias voltage V_ds_ due to the increase in carrier drift velocity and related reduction of the carrier transit time. We also recorded the dependence of I_ds_ on the back-gate voltage (V_g_) at a fixed V_ds_ of −10 V (Figure 7d). In the dark state, our device showed the typical behavior of a field-effect transistors with a p-type channel (black curve in Figure 7d), but when illuminated by the white light source, the OFF current increased from ~0.8 pA to ~14 pA, with the I_ds_ increasing at both OFF and ON states for all values of V_g_ (red curve in Figure 7d). This result indicates that the photocurrent dominated the thermonic and tunneling currents over the entire operating range of the device. According to the photocurrent generated under different V_g_, the responsivity (R = I_ph_/P_light_) was calculated and plotted as a function of V_g_ (blue curve in Figure 7d). Under a fixed V_ds_ of −10 V, the responsivity of the 2D GaSe crystal was ~1.7 A/W at zero gate voltage, and it increased to 8.5 W/A at a gate voltage of approximately −60 V, indicating that the back gate plays an important role in tailoring the photocurrent in these crystals. The responsivity from our vapor phase grown GaSe 2D crystals is comparable to that of exfoliated few-layered GaSe nanosheets (2.8 A/W)^23^ and higher than previously reported vapor-phase grown 2D GaSe (17 mW/A)^24^. The low or lack of electrical response for monolayer of GaSe is consistent with the declined in Raman intensities for monolayer GaSe. The results suggest that monolayer GaSe might not be ideal for some optoelectronic properties due to tradeoffs between dimensional confinement and a transition from a direct to indirect bandgap. The detailed correlations between layer number and their optoelectronic properties are being studied.

In summary, large, single crystalline, uniform monolayer and multi-layer GaSe crystals were grown on SiO_2_/Si substrate through a vapor-phase growth method. The size, density, shape, thickness, and uniformity of the crystals were shown to be controllable during the synthesis process. The multiscale structural characterizations show the synthesized 2D GaSe crystals have highly crystallinity, distinct crystal orientation, and clear grain boundary. The theoretical modeling of the electronic structure and Raman spectroscopy demonstrate a direct-to-indirect bandgap transition and progressive confinement-induced bandgap shifts for 2D GaSe crystals. The 2D GaSe crystals show a high photoresponse and FET characteristics, which demonstrate the potential of this material for electronic and optoelectronic applications such as FETs and photodetectors. The successful synthesis of large, uniform monolayer and multi-layer GaSe crystals on SiO_2_/Si substrate demonstrates that our method can be used potentially for the growth of other semiconducting metal monochalcogenides, e.g., GaS, GaTe, InS, InSe, and InTe, further enriching the building blocks for the fabrication of 2D electronic and optoelectronic devices.

## Methods

### Synthesis of bulk GaSe crystals

The synthesis was carried out in a tube furnace system equipped with a 2″ quartz tube. Ga_2_Se_3_ (99.99%, Alfa Aesar) and Ga (99.99%, Alfa Aesar) powders were mixed at a molar ratio of 1:1 and place at the center of the quartz tube. The tube was sealed and evacuated to ~5 × 10^−3^ Torr. The furnace was then heated to 950°C (from 25 to 700°C in 35 min and 700 to 950°C in 25 min) and maintained at temperature for 30 min. During synthesis, argon gas was used as a protective gas at 50 sccm, and the pressure was maintained at 300 Torr. Following growth, the furnace was cooled naturally to room temperature.

### Growth of 2D GaSe crystals

The synthesis of 2D GaSe was carried out in a tube furnace system equipped with a 1″ quartz tube (Figure S1). Bulk GaSe crystals and Ga_2_Se_3_ powder were mixed together (GaSe:Ga_2_Se_3_ molar ratio ~50/1), and were used as source materials. SiO_2_ (~300 nm)/Si pieces (1 × 1 cm^2^) were cleaned with acetone, isopropyl alcohol (IPA), and DI water, and used as growth substrates. In a typical run, ~60 mg of source powder and a piece of SiO_2_ (~300 nm)/Si substrate were loaded on a quartz boat, and subsequently inserted into the furnace. The source was located at the center of the furnace, with the substrate located ~8–13 cm downstream. After evacuating the tube to ~5 × 10^−3^ Torr, the reaction was conducted at 750°C (with a ramping rate of 20°C/min) for 2–10 min at a pressure of 30 Torr and an argon carrier gas flow rate of 50–100 sccm (standard cubic centimeter per minute). The vapor-phase reactants were transported by the flowing argon gas to the growth region, in which the temperature was ~650–720°C, thereby feeding the growth of the 2D GaSe crystals. After growth, the furnace was cooled naturally to room temperature.

### Fabrication of 2D GaSe devices

Electron beam lithography (FEI DB-FIB with Raith pattern writing software) was used for 2D GaSe FET fabrication. First, a layer of PMMA 495A4 was spun-coat on the 2D GaSe crystals, followed by a 180°C bake. After pattern writing and development, a 5 nm layer of Ti followed by a 30 nm layer of Au was deposited using electron beam evaporation. Finally, well-defined source and drain electrodes were revealed using lift-off process with Acetone/IPA.

### Characterization and theoretical calculation

The morphologies of the 2D GaSe crystals were characterized using optical microscopy (Leica DM4500 P), scanning electron microscopy (SEM; Zeiss Merlin SEM), and atomic force microscopy (AFM; Bruker Dimension Icon AFM). The composition was analyzed using energy-dispersive x-ray spectroscopy (EDS) within the SEM.

The crystal structures of the 2D GaSe were investigated using transmission electron microscopy (TEM), dark-field TEM (DF-TEM), and aberration-corrected scanning transmission electron microscopy (ADF-STEM). TEM imaging and diffraction were conducted using an FEI Technai T12 at 100 kV at low dose densities, and no detectable damage was observed during imaging. Acquisition times for DF-TEM images (both displaced-aperture and centered DF-TEM) were 2 s per frame. The ADF-STEM image was obtained using an aberration-corrected Nion UltraSTEM operating at 60 kV, using a half-angle range of the ADF detector from 86 to 200 mrad. The samples for TEM and STEM analysis were grown directly on amorphous silicon films (5 nm in thickness) supported by silicon TEM grid using the same growth process as described above.

Raman measurements were performed using cw excitation at 532 nm under a microscope (Jobin Yvon Horiba, T6400) using a long distance objective (100×, N/A = 0.8). The spot size on the sample was ~1 μm. During Raman mapping, the laser energy at the sample was maintained at ~1 mW, and the acquisition time was minimized to ~1 s for each 1 μm step to prevent photo-degradation of the sample.

The electrical properties and photoresponse of 2D GaSe devices were measured in vacuum under a probe station using a semiconductor analyzer (Keithley 4200) and a laser driven white light source. The dark state was measured without white light illumination, and it showed p-type semiconducting behavior when the gate voltage was swept at V_ds_ = −10 V. The power intensity of white light source was measured to be 1.2 mW/cm^2^ from 400 to 800 nm, and it was used for both the electrical and photoresponse measurements.

The electronic band structures of 2D GaSe crystals were investigated using a highly accurate, all-electron first-principles quantum mechanical calculation code (FHI-aims). The exchange-correlation potential of the Perdew-Burke-Ernzerhof (PBE) version of the generalized-gradient approximation (GGA) was used. For k-point samplings, 11 × 11 × 6 mesh points were used for bulk and 11 × 11 × 1 for the films with a large size vacuum (~50 Å). Different phases of bulk GaSe structures were modeled, such as *ε*, *β*, *δ*, and rhombohedral phases using experimental lattice parameters. All the bulk phases except the rhombohedral structure essentially have very similar electronic features such as bandgap energy, where valance band maximum (VBM) and conduction band minimum (CBM) are located at the G point. On the other hand, the rhombohedral structure shows an indirect bandgap with significantly larger bandgap energy to other phases by ~1 eV. Considering its large bandgap, we believe that the rhombohedral structure is not the observed structure in our experiments. We modeled multi-layered configurations of *ε*-GaSe to systematically study the changes in the electronic properties in terms of layer numbers.

## Author Contributions

K.X., X.F.L., C.M.R. and D.B.G. designed the experiments. X.F.L. synthesized the materials and performed optical microscopy, SEM, AFM characterizations. A.A.P. developed growth mechanisms and performed micro-Raman and PL spectroscopy measurements. J.C.I. performed the ADF-STEM characterization. M.F.C. and M.C. performed TEM and DF-TEM characterization. M.W.L. and I.I.K. fabricated the device and performed electrical and photoresponse measurements. M.Y. performed theory and modeling. X.F.L. and K.X. wrote the manuscript. All authors contributed to the discussion and final manuscript.

## Supplementary Material

Supplementary Information supplementary info
