# Supplementary material for: Controlled Vapor Phase Growth of Single Crystalline, Two-Dimensional GaSe Crystals with High Photoresponse
Source: Sci Rep. 2014 Jun 30;4:5497. doi: 10.1038/srep05497 (PMC4074793; doi:10.1038/srep05497)
Supplement: Supplementary Information — supplementary info [file srep05497-s1.pdf]

Supporting Information

Controlled Vapor Phase Growth of Single Crystalline, Two-Dimensional GaSe Crystals with High Photoresponse

*Xufan Li, Ming-Wei Lin, Alexander A. Puretzky, Juan C. Idrobo, Cheng Ma, Miaofang Chi, Mina Yoon, Christopher M. Rouleau, Ivan I. Kravchenko, David B. Geohegan, Kai Xiao**

Center for Nanophase Materials Sciences, Oak Ridge National Laboratory, Oak Ridge, TN, 37831, USA.

*To whom correspondence should be addressed: E-mail: xiaok@ornl.gov

Supplementary figures

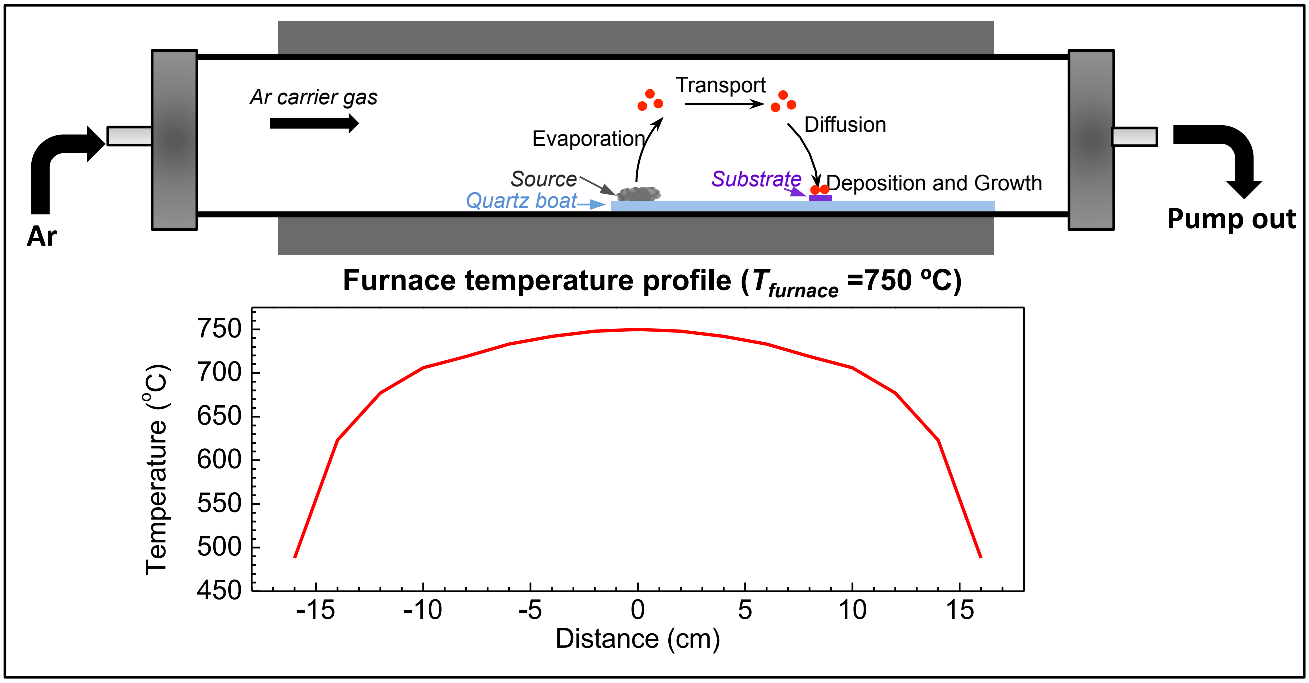

Figure S1. Top: Schematic diagram of a tube furnace system and the growth process of 2D GaSe crystals. **Bottom**: Temperature profile along the quartz processing tube when the furnace temperature is set at 750 °C. The temperature was measured using a thermocouple.

As illustrated in Figure S1, the growth generally contains the following steps: evaporation and transport of source materials by a flow of argon carrier gas, diffusion of source material vapor from the gas phase and deposition to the substrate, migration of adsorbed species to growth sites, growth of the crystals by incorporation of source species.

The Reynolds number (Re_x), which characterizes the flow of fluid, is calculated by the equation: $Re_x = \rho_g v_g d/\mu_g$, where ρ_g, v_g, and μ_g are density, mean velocity, and dynamic viscosity of the gas flow, respectively, and d is the diameter of the tube. The mean velocity, v_g, of 50 sccm argon gas under 30 Torr and at 750 °C is calculated to be ~3.6 m/s. Therefore, the Re_x value is calculated to be ~2.2. The value indicate that the gas flow in our growth is a laminar flow.

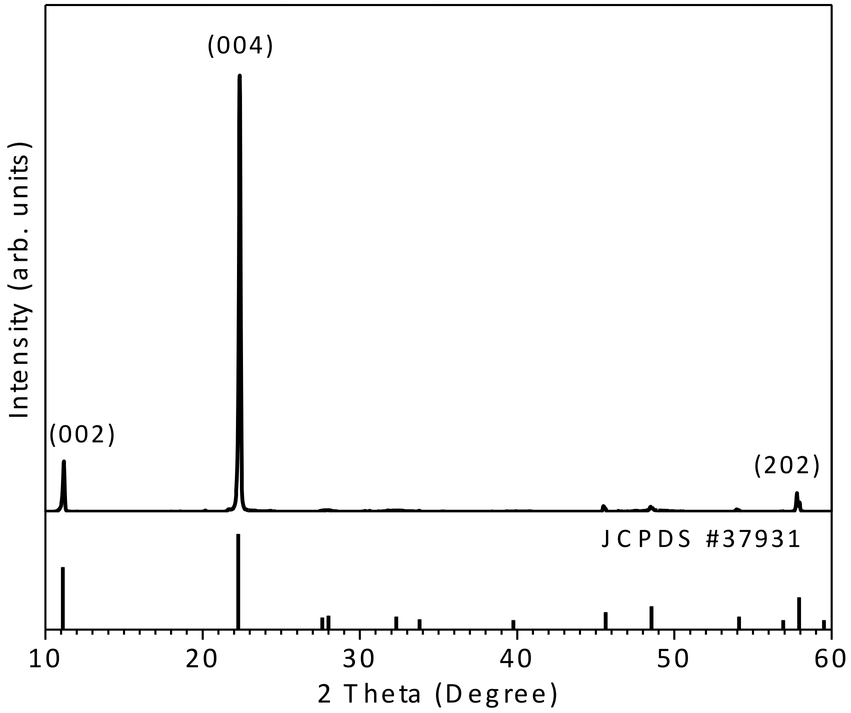

Figure S2. Powder x-ray diffraction pattern (XRD) obtained from bulk GaSe crystals with Cu Kα_1 radiation (λ = 1.5406 Å). The tick marks below the XRD pattern indicate the diffraction peak positions of hexagonal GaSe according to JCPDS #74-0794. The dominant (004) and (002) diffraction peaks in the XRD pattern indicates that the layered GaSe structure was preferentially stacked along the *c*-axis.

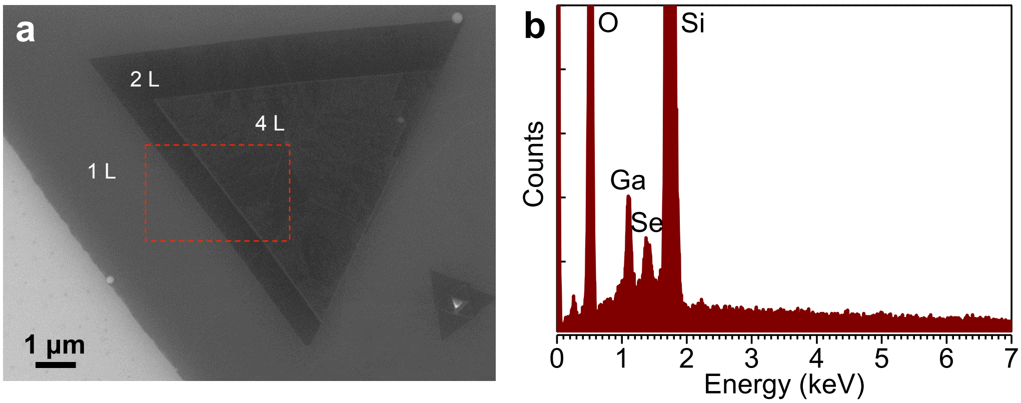

Figure S3. (**a**) SEM image of multi-layer GaSe crystals grown on a monolayer GaSe flake. (**b**) EDS spectrum obtained from the area included in the dashed-line square in (a). The atomic ratio of Ga:Se is 1:1 as calculated from the spectrum.

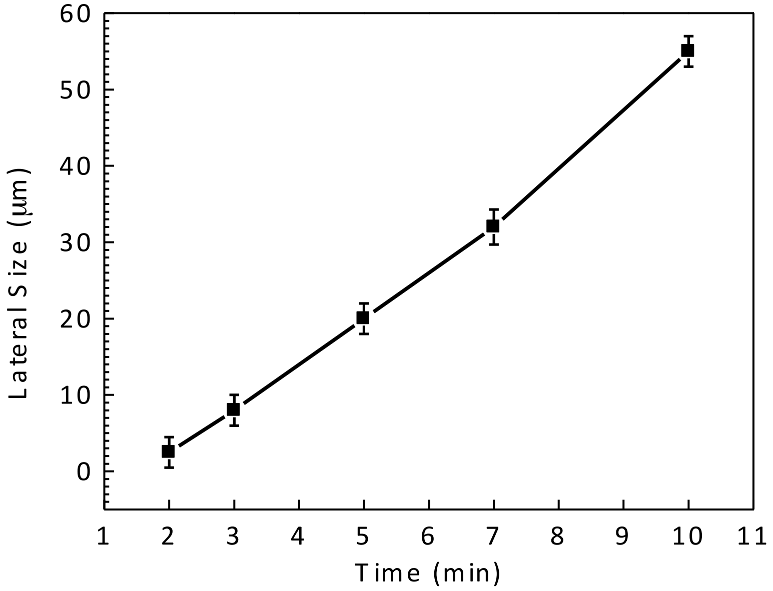

Figure S4. Lateral size of triangular monolayer GaSe flakes depending on growth times. When the growth time exceeds 10 min, almost all the monolayer flakes are merged to form continuous films, and therefore, it hard to distinguish individual triangles.

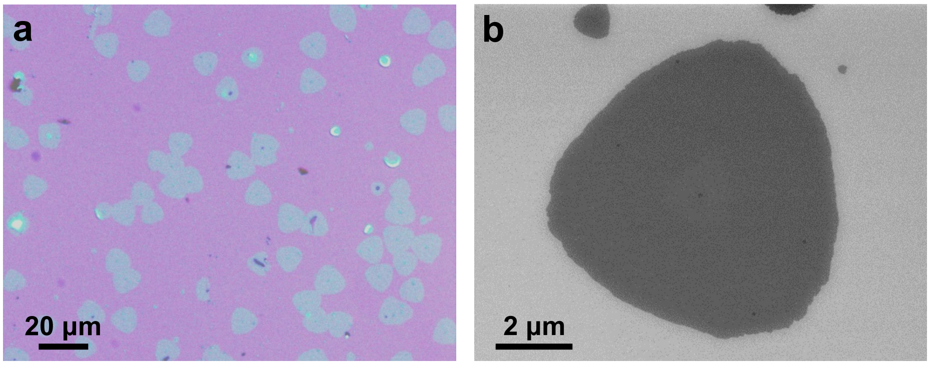

Figure S5. Morphologies of monolayer 2D GaSe crystals synthesized with only bulk GaSe crystals as the source. (**a**) Optical micrograph obtained near the downstream side of the substrate. (**b**) SEM image of an individual flake.

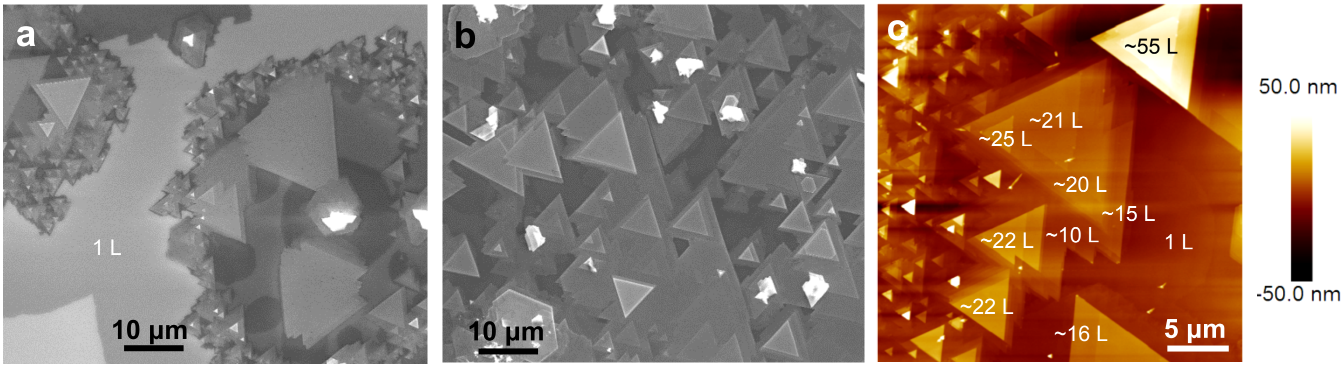

Figure S6. Morphologies of very thick multi-layer GaSe crystals. (**a**−**c**) Optical microscope images. The three images were obtained from three different areas on the substrate as indicated in Figure S1, which are close to the right (downstream) side (a) , middle (b) , and left (upstream) side (c) of the substrate. (**d**−**e**) SEM images. The monolayer GaSe on which thick multi-layer GaSe crystals are grown is shown in (e). (**f**) AFM image showing multi-layer GaSe crystals (from ~10 to ~55 L) grown on monolayer GaSe.

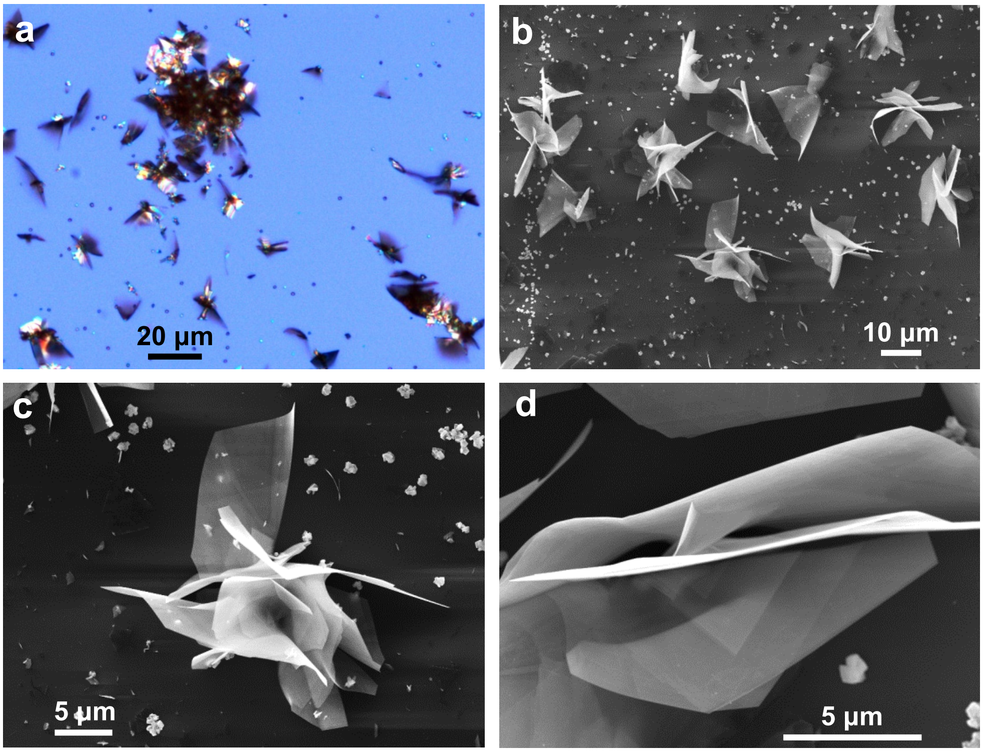

Figure S7. Morphologies of GaSe nanosheets grown out-of-plane from the substrate. (**a**) Optical micrograph. (**b**–**d**) SEM images.

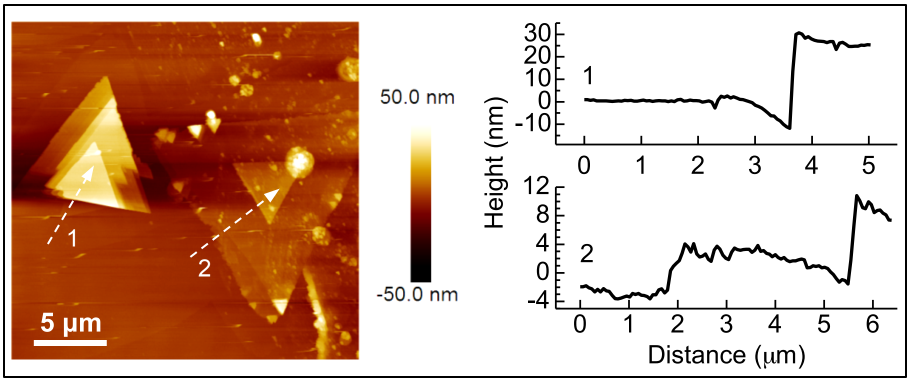

Figure S8. AFM images and line profiles of the 2D GaSe crystals for Raman and PL measurements shown in Figure 5a.

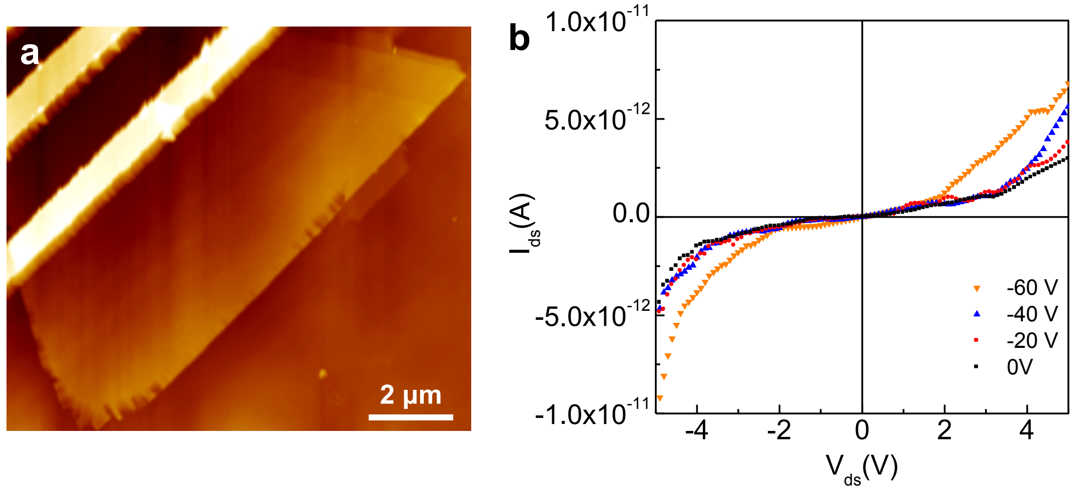

Figure S9. (**a**) AFM image with line profile of the 2D GaSe device as shown in Figure 7b. (**b**) Field-effect transistor output characteristics of the 2D GaSe device.
